# Chronic Conditions and Receipt of Treatment among Urbanized Rural Residents in China

**DOI:** 10.1155/2013/568959

**Published:** 2013-08-13

**Authors:** Juan Chen

**Affiliations:** Department of Applied Social Sciences, The Hong Kong Polytechnic University, Hung Hom, Kowloon, Hong Kong

## Abstract

While undergoing the unprecedented urbanization process in the past few decades, China has also experienced a major epidemiological shift from predominantly infectious diseases to chronic conditions. Using data from a national survey of 1,288 respondents in urban China, this study examines the prevalence of chronic conditions and receipt of treatment among urbanized rural residents who have experienced in situ urbanization. Negative binomial and logistic regressions were applied to estimate the differences in chronic conditions, receipt of treatment, and concern of seeking medical treatment among urbanized rural residents as compared to urban residents and rural-to-urban migrants. The results indicate that urbanized rural residents have similar number or prevalence of chronic conditions with urban residents, but they are less likely to receive treatment particularly for cardiovascular conditions. The analysis further reveals that urbanized rural residents are more anxious about their potential inability to cover medical expenses than both urban residents and rural-to-urban migrants. The study stresses the converging prevalence of chronic conditions but the continuing divide in receipt of treatment between urban residents and urbanized rural residents. As China's urbanization continues with the epidemiological transition, there is an urgent need to address such disparities.

## 1. Introduction

In the past three decades, China has not only seen the largest human migration in history but also an accelerated process of urbanization [1, 2]. The expansion of Chinese cities has been dramatic; the urban population jumped from 17.9% of the total population in 1978 to 51.3% in 2011 [3]. The scale and speed of China's urban growth has been primarily driven by rural industrialization, conversion of farmland, and rural-to-urban migration [2, 4]. Of the 440 million people who account for the urban growth since 1979, about half are temporary rural-to-urban migrants, whereas the rest are in situ “urbanized rural residents.” That is, more than 200 million new urbanites have actually never left their home village. Rather, the city came to them, either through relabeling their rural address as a city district or by rapidly expanding into the countryside that surrounded the villages [1, 3].

While undergoing the dramatic economic and social changes in the past few decades, China has also experienced a major epidemiological transition from predominantly infectious diseases to chronic conditions. Chronic, noncommunicable diseases are the leading causes of illness and death [5, 6]. In 2008, 270 million Chinese people were diagnosed with at least one chronic condition. Chronic, noncommunicable diseases account for an estimated 82% of total deaths and 70% of disability-adjusted life years lost. It is further predicted that the number of deaths attributable to chronic diseases will continue to rise to 85% by 2020 [7]. Chronic illness has become a major threat and burden to public health in China.

Studies that compare the health status of urban and rural residents indicate that although the prevalences of two-week illnesses and chronic diseases are significantly lower among the rural population, the primary causes of death among urban and rural populations have converged during the past two decades; cancer, stroke, heart disease, and respiratory disease are the top four causes of death in both cities and the countryside [8]. Previous studies on internal migration and health have consistently demonstrated the healthy migrant phenomenon; rural-to-urban migrants have a better self-reported health status and lower incidence of acute illnesses, chronic diseases, and disabilities than urbanites, even after controlling for age and education [9, 10]. Yet research on the health situation of those rural residents whose rural residence underwent in situ urbanization is scarce [11]. It is not clear whether these urbanized rural residents still keep the health advantage as former villagers or are catching up the prevalence of chronic diseases as new urbanites.

The cost of treating chronic disease continues to grow high in China [6]. High medical costs may deter patients from seeking necessary treatment. Many studies have examined Chinese use of health services, but few focus on treatments for chronic conditions in particular. Studies reveal that health service utilization for acute illnesses declined since the 1990s. In 1993, 59% of urban residents who were ill in the last two weeks visited a physician; the figure dropped to 50% in 1998, and to 43% in 2003 [12]. Rural residents are even less likely to receive treatment. In 2003, approximately 13% of urban residents and 19% of rural residents in need of outpatient care did not seek it due to increases in fees and low insurance coverage [13]. Seeking medical treatment is not common among the migrant population [14]. Peng et al. [15] reported that about 30% of migrant workers in Beijing who reported illness during the past two weeks did not seek any help, and nearly 20% of migrants requiring hospitalization failed to receive treatment within the past year. As chronic conditions usually require longer and continuing treatment than acute illnesses, a reluctance to seek medical treatment is likely to further deteriorate the situation. No study, however, has examined the use of health service among urbanized rural residents, not to mention the receipt of treatment for chronic conditions in particular.

Experience of urbanization can have both positive and negative consequences for individual health outcomes. On the one hand, urbanization offers opportunities for improvement in residents' health through better access to health care and service infrastructures; on the other hand, urban expansion also leads to environmental pollution and lifestyle changes that are detrimental to physical and mental health [16–20]. The uncontrolled expansion of Chinese cities has rapidly devoured surrounding rural areas. Between 1981 and 1999, the annual expansion of urban built-up areas averaged 800 square kilometers per annum. After 2000, the growth rate was doubled to more than 1,700 square kilometers per annum [2]. The total urban built-up area in 2011 was 43,603 square kilometers, almost six times of that in 1981 [21]. As urban boundaries expand, rural villages are subsumed. In large cities, the radial expansion of built-up areas produces “urban villages”—transitional neighborhoods characterized by insecure land rights and a mixture of rural and urban society [22]. Due to the rapidity of urbanization, in many rural areas that have become urban districts, the residents' *hukou* (household registration) status has not been changed, leaving them unable to receive the social benefits associated with urban *hukou*. Some of these residents do not have health insurance; others are still enrolled in the New Rural Cooperative Medical Scheme. The prevalence of chronic conditions and receipt of treatment among such residents is likely to follow a different pattern and therefore require research and policy attention.

Using data from a 2011 national survey of urban China, this study addresses two questions: First, what is the prevalence of chronic conditions of urbanized rural residents, and how does it differ from the rate of urban residents and rural-to-urban migrants? Second, what is the prevalence of receiving treatment for chronic conditions among urbanized rural residents, and how does it compare with the situation of urban residents and rural-to-urban migrants? To the best of our knowledge, this is the first study examining the health status of in situ urbanized rural residents who account for significant percentage of the new urbanites in China.

## 2. Methods

### 2.1. Sample and Data Collection

Data for this study come from the Migration and Quality of Life survey undertaken in May and June of 2011. Using spatial probability sampling specifically designed technology to reach urban residents regardless of their *hukou* status [23], we first selected 26 primary sampling units (PSUs), and then within each PSU, we selected 2 secondary sampling units (SSUs) in areas where the average nighttime light was higher than 30 on a scale of 0–63 per pixel based on the Operational Linescan System nighttime light data provided by the Defense Meteorological Satellite Program (OLS-DMSP) in 2009. We chose a threshold of 30 to define the sample frame of physical areas deemed “urban” iteratively which pretty much matches with the urban areas according to the national standard definition [24]. From these 26 PSUs and 52 SSUs that spread over 19 provinces, 27 prefectures, and 31 counties or city districts, we sampled a total of 1,906 households and successfully interviewed 1,288 individuals between the ages of 18 and 70 who were randomly selected from each household according to the Kish grid method. All interviews were conducted in person by trained interviewers. The average length of the interviews was 38.3 minutes. The response rate was 67.6%. To ensure quality control, a random sample of the participants was called back to validate the data. Ten cases were excluded due to missing data, leaving a sample of 1,278. The analysis applied weights and employed the “svy” (survey) commands in Stata 11.0 to account for the survey design effects.

### 2.2. Measures

Chronic health conditions were assessed with the World Mental Health Composite International Diagnostic Interview (WMH-CIDI). Respondents were asked if they had any of the listed physical and psychophysiological disorders in their lifetime and during the past 12 months. The total number ranges from 0 to 10. The conditions were further divided into four classifications: pain-related, cardiovascular, respiratory, and others. Each of these four categories was coded “1” if the respondent had any of the conditions and “0” if not. Respondents reported whether they received any treatment for each of the chronic conditions during the past 12 months (1 = yes; 0 = no). Respondents were also asked about their concern of seeking medical treatment: “Do you worry about paying medical bills when you have a serious illness?” (1 = very much; 0 = somewhat or not at all).

Migration and residency status was coded into urban residents (those with urban *hukou*), rural-to-urban migrants (those with rural *hukou* but residing in an urban area rather than their registered address), and urbanized rural residents (those with rural *hukou* residing in an urban area and at their registered address). Demographic information included age, gender, and marital status. Measures of socioeconomic status were education, occupation, and household wealth (an index based on ownership of a number of consumer items, such as a television and car). Health insurance included three dichotomous variables: urban basic medical insurance, New Rural Cooperative Medical Scheme, and commercial health insurance.

### 2.3. Analysis

Descriptive statistics were first computed among urban residents, rural-to-urban migrants, and urbanized rural residents, respectively (Table 1). Negative binomial regressions were then estimated on the number of lifetime, and 12-month chronic conditions and logistic regressions were estimated on the prevalence of pain-related conditions and cardiovascular conditions. Respondents' sociodemographic characteristics and migration and residency status were included as the independent variables (Table 2). Logistic regressions were further applied to estimate the differences in receipt of treatment in the past 12 months according to migration and residency status controlling for sociodemographic characteristics among those with any or specific chronic health conditions (Table 3). The models were estimated solely for chronic conditions, pain-related conditions, and cardiovascular conditions, not for respiratory conditions or other conditions because their prevalence rates are too low to make any meaningful inferences. Finally, logistic regressions were employed to model the associations between respondents' concern of seeking medical treatment and migration and residency status, controlling for sociodemographic characteristics and health insurance (Table 4).

## 3. Results

### 3.1. Descriptive Statistics

Table 1 reports the descriptive statistics of both lifetime and 12-month chronic conditions among urban residents, rural-to-urban migrants, and urbanized rural residents. On average, the number of lifetime chronic conditions for urban residents and urbanized rural residents is similar, 0.69 and 0.73, respectively, while rural-to-urban migrants reported a significantly lower number 0.23. About 36 to 37% of urban residents and urbanized rural residents have at least one chronic condition, and about 19 to 20% of them have more than one chronic condition; these prevalence rates are 2 to 3 times higher than those among rural-to-urban migrants (16% and 6%, resp.). For each specific condition, about one-third of urban residents and urbanized rural residents but only 13% of rural-to-urban migrants reported pain-related conditions; urban residents reported a much higher prevalence of cardiovascular conditions (10%) than rural-to-urban migrants and urbanized rural residents (2–5%); the prevalence of respiratory and other conditions is relatively low among urban residents and urbanized rural residents (from 0.23 to 3.28%) and extremely low among rural-to-urban migrants (from 0.03 to 0.92%). On measures of 12-month chronic conditions, we observe the same pattern among the three groups but slightly lower numbers and prevalence rates. The results are consistent with the healthy migrant phenomenon for rural-to-urban migrants but indicate no significant difference between urbanized rural residents and urban residents.

### 3.2. Regressions Results on Number and Prevalence of Chronic Conditions

Table 2 presents the coefficients (with standard errors) from the negative binomial regressions on the number of lifetime and 12-month chronic conditions and the odds ratios (with 95% confidence intervals) from the logistic regressions on the prevalence of pain-related conditions and cardiovascular conditions. After controlling for sociodemographic characteristics, the number and prevalence of chronic conditions still follow the same pattern, that is, no significant difference between urban residents and urbanized rural residents either during the lifetime or in the past 12 months. The healthy migrant phenomenon is also observed, except on the prevalence of lifetime cardiovascular conditions.

### 3.3. Regressions Results on Receipt of Treatment for Chronic Conditions

Table 3 includes the odds ratios (with 95% confidence intervals) from the logistic regressions estimating the differences in receipt of treatment in the past 12 months among different migrant and resident groups. Because the prevalence rates of respiratory and other conditions are too low (as shown in Table 1) to make any meaningful inferences, models were only estimated among respondents with any pain-related and cardiovascular conditions. Controlling for demographic characteristics, urbanized rural residents are less likely to receive treatment than urban residents for any chronic conditions (odds  ratio = 0.27, *P* < 0.01) and for cardiovascular conditions in particular (odds  ratio = 0.02, *P* < 0.01). When measures of socioeconomic status are further controlled, urbanized rural residents are still significantly less likely to receive treatment than urban residents for cardiovascular conditions (odds  ratio = 0.00, *P* < 0.001); the odds ratio for any chronic condition is similar (0.30) but statistically insignificant. Similar to urbanized rural residents, rural-to-urban migrants are also less likely to receive treatment for cardiovascular conditions than urban residents, no matter whether socioeconomic status is controlled. There is no significant group difference in receipt of treatment among those with pain-related conditions.

### 3.4. Regression Results on Concern of Seeking Medical Treatment

Table 4 reports the logistic regression results estimating the differences in concern of seeking medical treatment according to migration and residency status, controlling for number of chronic conditions or prevalence of specific conditions, sociodemographic characteristics, and health insurance. The results indicate no significant difference between rural-to-urban migrants and urban residents, yet urbanized rural residents are two times more likely to worry about paying medical bills for serious illnesses (in Model 4, odds  ratio = 1.96, *P* < 0.01). Pain-related and respiratory conditions are significant predictors of the concern. None of the health insurance schemes significantly lessen the concern.

## 4. Discussion

Based on data from a national survey of a representative sample of China's urban population, this study for the first time documents the prevalence of chronic conditions and receipt of treatment among those urbanized rural residents who have experienced in situ urbanization as compared to urban residents and rural-to-urban migrants. The results confirm the healthy rural-to-urban migrant phenomenon except on the prevalence of lifetime cardiovascular conditions, but they indicate no difference between urbanized rural residents and urban residents in the average number or prevalence of chronic conditions either during the lifetime or in the past 12 months. The analysis further demonstrates that compared with urban residents, both rural-to-urban migrants and urbanized rural residents are less likely to receive treatment for cardiovascular conditions; however, urbanized rural residents are also less likely to receive treatment for any chronic conditions before controlling for socioeconomic status. In addition, urbanized rural residents are most anxious about their potential inability to cover medical expenses, and none of the health insurance scheme reduces this concern of seeking medical treatment.

The prevalence of chronic conditions and the narrowing gap between long-term urban residents and recently converted urbanites, as opposed to the healthy migrant phenomenon, provide us a better understanding of the separate effects of migration and urbanization on health. While the city is drawing 200 million healthy young adults out of the countryside, the city is also rapidly expanding into the countryside and converting 200 million rural residents into urbanites. These urbanized residents are passively merged into the urban life. The findings in this study suggest that, different from those young migrant adults, the urbanized rural residents are not keeping any health advantage as former villagers, but rather catching up the prevalence of chronic diseases as new urbanites. The potential risks of urbanization of the world's most populous nation, including the loss of arable land, the creation of poor urban enclaves, and the deterioration of residents' health outcomes, require a great deal of research and policy attention.

As China's rapid urbanization proceeds at unprecedented scale and pace, the health and economic implications of chronic conditions are likely to be huge and present tremendous challenges. The situation is particularly serious in China where family support has become more precarious because of the one-child policy [25]. The government's ambitious plans to expand health insurance enrolment and health care coverage do achieve impressive results [26]. The wide coverage offered by both the urban and the rural basic health insurance schemes is encouraging; however, the urban-rural divide in health insurance coverage based on *hukou* is still pronounced. The data for this study show that 62% of urban residents are covered by the urban basic medical insurance, while 80% of urbanized rural residents are enrolled in the New Rural Cooperative Medical Scheme. The majority of rural-to-urban migrants are also in NRCMS. Commercial health insurance is popular only among urban residents.

The health care access among the group of urbanized rural residents requires further research and policy attention. They are the original rural *hukou* holders residing in a recent urbanized place. It is likely that during the rapid urbanization process, the area has been converted or merged into an urban district, but the residents there have not changed their *hukou* status from rural to urban, nor have they acquired the associated social benefits. During the urbanization process, efforts must be made to ensure their *hukou* transition and access to the urban health care system. Meanwhile, the results of this study show that socioeconomic status, particularly professional or managerial occupation, is an important predictor for receiving treatment for chronic conditions. Resources and employment opportunities therefore need to be promoted to enhance the livelihood and socioeconomic status of urbanized rural residents.

Furthermore, the efficacy of the existing health insurance schemes in providing treatment for chronic conditions is limited. The findings in this study suggest that belonging to the urban or the rural basic health insurance scheme does not reduce the concern about potential inability to cover medical expenses. Other scholars have also observed that NRCMS offers very limited protection in type of care and reimbursement of health care costs [27–29]. Having health insurance is critical, but it must be an insurance with an adequate range of coverage and level of protection [30, 31]. It is therefore necessary not only to reduce the urban-rural divide in health insurance coverage, but also to scale up NRCMS as well as the urban basic health insurance so that they can cope with the increasing medical expenditure for chronic diseases.

A few limitations should be noted. First, the study focuses on chronic conditions. These health conditions are not always verified by clinical diagnosis and do not represent incident cases. Nonetheless, this measure of health has been used in prior studies and correlated with clinical measures of morbidity [32]. Second, the analysis does not compare the efficacies of different types of health insurance in promoting receipt of treatment for chronic conditions because of the concern of endogeneity based on cross-sectional data. Further work is needed to assess the likelihood of success of the health care reforms in promoting treatment for chronic conditions based on longitudinal follow-ups.

Despite the above limitations, this study represents a timely investigation of the prevalence of chronic conditions and receipt of treatment among the group of urbanized rural residents who have experienced in situ urbanization in China. The research stresses the converging prevalence of chronic conditions between long-term urban residents and these new urbanites yet the continuing urban-rural divide in health insurance based on *hukou*, as well as the anxiety held by urbanized rural residents in paying medical bills. As China's urbanization process continues along with the major epidemiological transition from predominantly infectious diseases to chronic conditions, the group of urbanized rural residents deserves more research and policy attention, and the issues they encounter must be tackled with an integrated approach.

## Figures and Tables

**Table 1:** Descriptive statistics by migration and residency status (*N* = 1,278).

	Urban residents	Rural-to-urban migrants	Urbanized rural residents
Chronic health conditions—lifetime			
Total number of chronic conditions (1 min, 10 max; mean)^c^	0.69 (0.22)	0.23 (0.08)	0.73 (0.08)
At least one chronic condition (%)^c^	36.22	15.67	37.26
More than one chronic conditions (%)^c^	18.99	6.01	19.57
Pain-related conditions (%)^c^	34.50	13.07	34.47
Cardiovascular conditions (%)	10.07	2.19	4.83
Respiratory conditions (%)	0.23	0.92	0.99
Other conditions (%)	3.28	0.03	1.41
Chronic health conditions—past 12 months			
Total number of chronic conditions (1 min, 10 max; mean)^a,c^	0.59 (0.16)	0.21 (0.07)	0.61 (0.11)
At least one chronic condition (%)^c^	34.47	13.79	32.92
More than one chronic conditions (%)^c^	14.12	5.87	15.38
Pain-related conditions (%)^c^	30.35	12.40	31.15
Cardiovascular conditions (%)^a^	7.47	0.87	3.77
Respiratory conditions (%)	0.16	0.92	0.99
Other conditions (%)	3.15	0.03	0.41
Received treatment during the past 12 months			
Among those with any 12-month chronic conditions (%)	90.46	82.37	75.37
Among those with 12-month pain-related conditions (%)	85.50	87.14	74.46
Among those with 12-month cardiovascular conditions (%)	99.40	84.47	90.83
Among those with 12-month respiratory conditions (%)^a,c^	82.54	13.15	100.00
Among those with 12-month other conditions (%)	98.61	100.00	98.51
Concern of seeking medical treatment			
Worry about paying medical bills (very much, %)^b,c^	11.95	6.78	25.93
Socio-demographic characteristics			
Age (years, mean)^a^	43.95 (2.02)	37.60 (3.31)	44.73 (3.08)
Gender (female, %)	53.62	49.74	44.81
Marital status (married, %)	88.12	80.16	85.94
Education (years of schooling, mean)^b^	10.46 (1.65)	7.72 (0.88)	6.64 (0.97)
Occupation (professional/managerial, %)	25.54	10.89	3.87
Household wealth (0 min, 12 max; mean)	6.46 (1.03)	5.65 (0.73)	5.51 (0.37)
Health insurance			
Urban basic medical insurance (%)^a,b^	62.16	8.91	2.55
New Rural Cooperative Medical Scheme (%)^a,b^	16.63	66.73	80.02
Commercial health insurance (%)^c^	23.80	4.01	9.04
Sample size *N *	613	231	434
Weighted percentage (%)	56.78	18.13	25.09

Survey design effects (strata, cluster, and individual weight) are adjusted in the mean estimations.

Means or percentages are reported; standard errors in parentheses.

^a^Difference between urban residents and rural-to-urban migrants significant at *P* < 0.05.

^b^Difference between urban residents and urbanized rural residents significant at *P* < 0.05.

^c^Difference between rural-to-urban migrants and urbanized rural residents significant at *P* < 0.05.

**Table 2:** Regressions on number and prevalence of chronic conditions: lifetime and past 12 months (*N* = 1,278).

	Lifetime			Past 12 months		
	Number of chronic conditions^a^	Pain-related conditions^b^	Cardiovascular conditions^b^	Number of chronic conditions^a^	Pain-related conditions^b^	Cardiovascular conditions^b^
Urban residents (reference group)						
Rural-to-urban migrants	−0.61*	0.47*	**0.48**	−0.61*	0.48*	0.23**
	(0.28)	[0.23, 0.93]	[0.11, 2.11]	(0.26)	[0.25, 0.92]	[0.09, 0.60]
Urbanized rural residents	**0.04**	**1.23**	**0.44**	**0.04**	**1.14**	**0.65**
	(0.19)	[0.46, 3.33]	[0.12, 1.59]	(0.24)	[0.43, 3.03]	[0.16, 2.60]
Age	0.08***	1.11***	1.17***	0.07***	1.08***	1.18***
	(0.01)	[1.06, 1.15]	[1.11, 1.23]	(0.01)	[1.05, 1.11]	[1.11, 1.25]
Female	0.05	1.18	1.14	0.04	1.11	0.67
	(0.11)	[0.75, 1.87]	[0.63, 2.06]	(0.11)	[0.69, 1.80]	[0.42, 1.08]
Married	−0.72*	0.20**	0.21**	−0.52	0.36	1.33
	(0.26)	[0.07, 0.58]	[0.08, 0.52]	(0.38)	[0.09, 1.38]	[0.17, 10.52]
Years of schooling	0.02	1.06	1.06	0.02	1.04	1.05
	(0.02)	[0.94, 1.19]	[0.96, 1.18]	(0.02)	[0.94, 1.14]	[0.94, 1.17]
Professional/managerial occupation	0.68**	1.63	2.96*	0.68*	1.40	8.43*
	(0.23)	[0.82, 3.26]	[1.20, 7.33]	(0.32)	[0.71, 2.75]	[1.06, 67.05]
Household wealth	0.02	1.06	1.22*	−0.03	1.00	1.04
	(0.04)	[0.80, 1.40]	[1.00, 1.47]	(0.05)	[0.79, 1.25]	[0.79, 1.39]
Wald *F* statistics	36.16 (8, 19)	41.10 (8, 19)	22.82 (8, 19)	36.37 (8, 19)	19.53 (8, 19)	31.17 (8, 19)

Survey design effects (strata, cluster, and individual weight) are adjusted in the model estimations.

^a^Negative binomial regressions are estimated. Coefficients are reported; standard errors in parentheses.

^b^Logistic regressions are estimated. Odds ratios are reported; 95% confidence intervals in brackets.

*P < 0.05, **P < 0.01, and ***P < 0.001.

**Table 3:** Regressions on receipt of treatment for any or specific chronic conditions in the past 12 months.

	Any chronic conditions (*n* = 433)	Pain-related conditions (*n* = 358)	Cardiovascular conditions (*n* = 130)	Any chronic conditions (*n* = 433)	Pain-related conditions (*n* = 358)	Cardiovascular conditions (*n* = 130)
Urban residents (reference group)						
Rural-to-urban migrants	**0.59**	**1.69**	0.03**	**0.51**	**1.45**	0.00***
	[0.14, 2.50]	[0.30, 9.42]	[0.00, 0.34]	[0.11, 2.43]	[0.25, 8.61]	[0.00, 0.01]
Urbanized rural residents	0.27**	**0.48**	0.02**	**0.30**	**0.51**	0.00***
	[0.10, 0.70]	[0.16, 1.43]	[0.00, 0.34]	[0.05, 1.68]	[0.10, 2.65]	[0.00, 0.01]
Age	1.06	1.04	1.18***	1.04	1.03	1.28**
	[1.00, 1.12]	[0.99, 1.10]	[1.08, 1.28]	[0.99, 1.09]	[0.98, 1.08]	[1.10, 1.49]
Female	1.00	1.64	1.49	0.75	1.29	1.85
	[0.59, 1.67]	[0.86, 3.15]	[0.20, 11.01]	[0.34, 1.65]	[0.61, 2.73]	[0.27, 12.80]
Married	0.64	0.80	1.45	0.72	0.85	6.46
	[0.10, 4.29]	[0.14, 4.47]	[0.12, 17.29]	[0.14, 3.62]	[0.17, 4.21]	[0.22, 186.72]
Years of schooling				0.86	0.86	1.19
				[0.65, 1.12]	[0.65, 1.15]	[0.93, 1.52]
Professional/managerial occupation				44.83**	92.50***	0.02*
				[3.23, 622.25]	[11.73, 729.50]	[0.00, 0.46]
Household wealth				1.15	1.12	0.60*
				[0.88, 1.51]	[0.82, 1.52]	[0.41, 0.89]
Wald *F* statistics	4.34 (5, 19)	2.28 (5, 19)	13.04 (5, 19)	4.62 (8, 19)	4.45 (8, 19)	6.12 (8, 19)

Survey design effects (strata, cluster, and individual weight) are adjusted in the model estimations.

Logistic regressions are estimated. Odds ratios are reported; 95% confidence intervals in brackets.

*P < 0.05, **P < 0.01, and ***P < 0.001.

**Table 4:** Regressions on concern of seeking medical treatment (*N* = 1,278).

	Model 1	Model 2	Model 3	Model 4
Urban residents (reference group)				
Rural-to-urban migrants	**0.57**	**0.61**	**0.52**	**0.56**
	[0.23, 1.44]	[0.22, 1.71]	[0.22, 1.24]	[0.21, 1.48]
Urbanized rural residents	1.96***	2.02***	1.86**	1.96**
	[1.47, 2.62]	[1.50, 2.71]	[1.29, 2.68]	[1.21, 3.18]
Age	0.98*	0.98*	0.98	0.98
	[0.96, 1.00]	[0.96, 1.00]	[0.96, 1.00]	[0.96, 1.00]
Female	1.03	1.03	1.03	1.03
	[0.65, 1.64]	[0.63, 1.70]	[0.63, 1.67]	[0.58, 1.83]
Married	3.01*	3.57**	3.39*	4.02**
	[1.20, 7.56]	[1.48, 8.62]	[1.33, 8.63]	[1.64, 9.83]
Years of schooling	1.00	0.99	1.01	1.00
	[0.89, 1.13]	[0.88, 1.11]	[0.90, 1.14]	[0.89, 1.13]
Professional/managerial occupation	0.42	0.47	0.56	0.59
	[0.02, 8.23]	[0.03, 8.68]	[0.03, 11.91]	[0.03, 12.69]
Household wealth	0.85	0.84*	0.89	0.89
	[0.72, 1.00]	[0.72, 0.98]	[0.75, 1.05]	[0.76, 1.04]
Number of chronic conditions	1.62**		1.66***	
	[1.23, 2.13]		[1.27, 2.17]	
Pain-related conditions		3.67**		3.89**
		[1.68, 7.99]		[1.75, 8.65]
Cardiovascular conditions		2.24		2.39
		[0.77, 6.48]		[0.84, 6.86]
Respiratory conditions		2.60*		2.57*
		[1.19, 5.68]		[1.09, 6.08]
Other conditions		0.27		0.30
		[0.07, 1.09]		[0.07, 1.35]
Urban basic medical insurance			0.57	0.53
			[0.29, 1.11]	[0.28, 1.02]
New Rural Cooperative Medical Scheme			0.73	0.67
			[0.40, 1.34]	[0.33, 1.38]
Commercial health insurance			0.41	0.42
			[0.05, 3.19]	[0.06, 2.85]
Wald *F* statistics	19.15 (9, 19)	34.24 (12, 19)	18.58 (12, 19)	26.04 (15, 19)

Survey design effects (strata, cluster, and individual weight) are adjusted in the model estimations.

Logistic regressions are estimated. Odds-ratios are reported; 95% confidence intervals in brackets.

*P < 0.05, **P < 0.01, and ***P < 0.001.
